# Cardiac MRI in post COVID acute myocarditis: A case report

**DOI:** 10.1016/j.idcr.2022.e01579

**Published:** 2022-07-19

**Authors:** Nirmal Prasad Neupane, Kritisha Rajlawot, Chandramani Adhikari, Devraj Kandel, Irfa Mustafa

**Affiliations:** a Department of Radiodiagnosis and Imaging, Shahid Gangalal National heart Centre, Bansbari, Kathmandu, Nepal; b Department of Cardiology, Shahid Gangalal National heart Centre, Bansbari, Kathmandu, Nepal; c Department of Radiodiagnosis and Imaging, Shahid Gangalal National heart Centre, Bansbari, Kathmandu, Nepal

**Keywords:** Cardiac Magnetic Resonance Imaging (CMR), Post coronavirus disease 2019 (COVID-19) myocarditis, T2- short-tau inversion recovery (STIR), Late Gadolinium Enhancement (LGE)

## Abstract

Myocarditis is an acute or chronic inflammatory reaction of the heart muscle frequently associated with viral infections and post-viral immune-mediated responses. Recently the SARS-CoV-2 virus has been identified as a cause of myocarditis in COVID-19 patients. The role of cardiac MRI in such patients hence has become a subject of concern. Thus, we present a case of post-COVID-19 myocarditis where cardiac MRI was helpful in establishing the diagnosis.

## Introduction

Myocarditis is an acute or chronic inflammatory reaction of the heart muscle that can be elicited by a multitude of viral, immune-mediated, or toxic factors [1]. Although the term "Myocarditis" clearly refers to inflammation of the myocardium, the clinical diagnosis is not always obvious. Firstly, myocarditis has a wide spectrum of clinical manifestations. Second, it is not feasible to perform an endomyocardial biopsy (EMB) on a routine basis due to its invasiveness though it is being considered the gold standard in diagnosing myocarditis [2]. Cardiac magnetic resonance (CMR) imaging, a non-invasive imaging modality, has already been introduced and established for non-invasively detecting myocarditis. Moreover, CMR features of myocarditis in patients post coronavirus disease 2019 (COVID-19) infection have recently become a subject of concern. In several cases of COVID-19, cardiovascular complications have been reported and the majority concluded myocarditis as the cause of primary heart dysfunction [3]. We hereby present a case of post-COVID-19 myocarditis where CMR was helpful in the early diagnosis and management of the patient.

## Clinical history

A female patient aged 45 years presented to our emergency department with complaints of acute chest pain, dyspnea, palpitation, and arrhythmia in the form of ventricular tachycardia. Her routine tests showed electrocardiographic changes with increased troponin and C-reactive protein levels. Given the possibility of having coronary artery disease based on her clinical scenario, she underwent CT coronary angiography which revealed normal coronary arteries with no evidence of thrombosis or occlusion. She revealed she had recently been diagnosed with COVID-19 two weeks prior. Therefore, the patient was referred for a CMR to look for other possible causes of her cardiac symptoms.

## Imaging findings

**Fig. 1:**
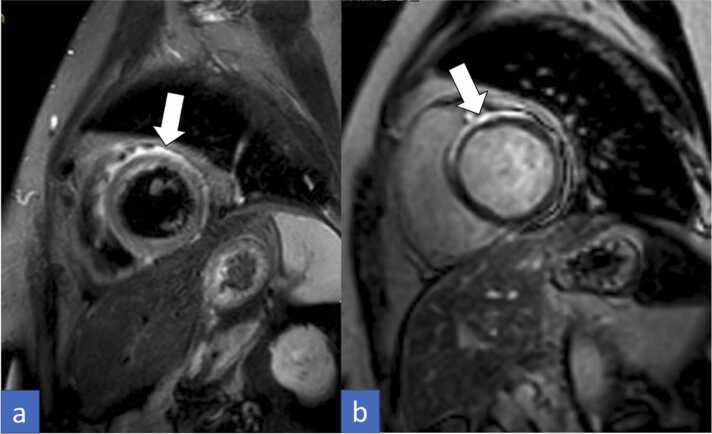
Cardiac Magnetic Resonance (CMR) T2 short-tau inversion recovery (STIR) (a) and post-contrast delayed gadolinium-enhancement (DGE) image short-axis view (b) showing high signal intensity in the myocardium with a subepicardial pattern of delayed GAD enhancement in the anterior and anteroseptal segments (arrow).

**Fig. 2:**
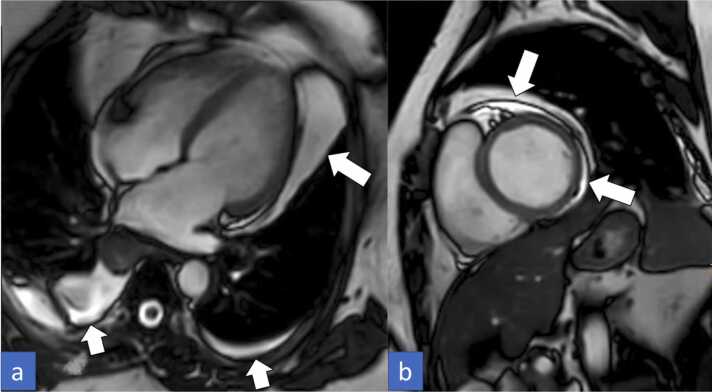
Cardiac Magnetic Resonance (CMR) bright blood images four-chamber view (a) showing minimal pericardial effusion (long arrow) and minimal to moderate bilateral pleural effusion (short arrows), short-axis view (b) showing minimal pericardial effusion (long arrows).

Cardiac MRI with contrast was performed for the patient on a 3Tesla platform. The CMR sequences of transverse black blood and bright blood images, vertical long axis, four-chamber, short-axis cine images, left ventricular outflow tract (LVOT) views along with three-chamber cine images were obtained. The short-tau inversion recovery (STIR) images were obtained from the base to the apex of the heart in the short axis. The delayed gadolinium enhancement phase-sensitive inversion recovery (PSIR) sequences were also obtained in short axis, four-chamber, and vertical long-axis views. The imaging findings showed a reduced left ventricular ejection fraction (LVEF) of 38% with hypokinetic anterior and anteroseptal segments of the basal and mid cavity. STIR images showed high signal intensity in the above-mentioned segments suggestive of edematous changes. The corresponding segments of the basal and mid cavity depicted subepicardial delayed gadolinium enhancement (DGE) in the post-contrast study (Fig. 1). Mild mitral and aortic regurgitation were also present with minimal pericardial effusion and bilateral pleural effusion, moderate on the right, and minimal on the left pleural cavity (Fig. 2). Hence, based on the CMR findings, the patient was diagnosed with acute myocarditis using modified Lake Louise Criteria (LLC).

## Discussion

Myocarditis- a nonischemic inflammatory disease of the myocardium, is frequently associated with viral infections and post-viral immune-mediated responses [2]. It may occur at any age but is more prevalent among young individuals which is one criterion our case meets. Among different viruses causing inflammatory changes in the cardiac muscle, the SARS-CoV-2 virus has been identified as a cause of cardiac impairment with about 30% of the COVID-19 patients experiencing cardiac injury [4]. Myocarditis encompasses a wide variety of non-specific clinical symptoms that may not be always helpful enough in determining its diagnosis. Here is where CMR plays a crucial role by aiding in characterizing myocarditis. CMR imaging has been used to assess myocarditis following Lake Louise criteria (LLC) since 2009, which mainly comprised of edema, hyperemia, and necrosis/or fibrosis. However, several limitations were gradually discovered, and the original LLC was modified in 2018. As per the 2018 LLC, the diagnosis of CMR-based myocarditis should include at least one T1-based criterion (increased myocardial T1 relaxation times, extracellular volume fraction, or Late Gadolinium Enhancement, LGE) and at least one T2-based criterion (increased myocardial T2 relaxation times, visible regional high T2 signal intensity representing edema on T2 STIR, or increased T2 signal intensity ratio) [1], [5], [6].

## CMR findings in post COVID myocarditis

### Myocardial edema

Post COVID myocarditis may show either localized or diffuse edematous changes which is a characteristic feature signifying inflammation. The myocardial edema on CMR is seen as a high T2 signal intensity or a global or localized increase in cardiac T2 relaxation time where T2 hyperintensity essentially indicates increased tissue water [7].

### DGE in myocardial injury

Myocardial injury is a cardinal feature of myocarditis and LGE imaging is considered among the most essential MRI sequences in the possible myocarditis. The injured or damaged myocardial tissue appears as a hyperintense area in the post contrast images representing the retention of contrast agents. Furthermore, the DGE sequence is also a reliable and useful marker to indicate the fatality associated with myocarditis [7]. DGE along with edema or T2 hyperintensity suggests a better prognosis as edema may resolve with time. On the other hand, DGE without associated edema usually indicates irreversible fibrosis and thus suggests a poor prognosis [7], [8].

Both of those CMR findings of myocardial edema and DGE with accompanied T2 hyperintensity were present in our case along with the associated ancillary features such as minimal pericardial effusion and minimal to moderate pleural effusion. CMR in post-COVID-19 patients may reveal a high incidence of diffuse myocardial edema, with a regional increase of T1 and T2 mapping values. However, the overall CMR characteristic features of myocarditis in post-COVID-19 patients and non-COVID-19 individuals may resemble following the globally recognized Lake Louis Criteria [7], [9]. Hence, in patients with COVID-19 infection who present with persisting chest tightness, chest pain, or breathlessness, CMR needs to be undertaken to rule out the possibility of acute myocarditis given that CMR is an established gold standard non-invasive modality to diagnose myocarditis [2]. CMR may thus be the modality of choice in individuals with COVID 19 infection suspected of having myocarditis as it enables the identification of areas of myocardial damage and further provides precise and accurate estimation of the cardiac function [10]. CMR, however, has its limitations too and might not be undertaken in every individual. It is a time-consuming procedure and is contraindicated to some extent in patients with pacemakers and defibrillators. In such cases, alternate imaging modalities can be used.

## Management

Regarding the clinical management of COVID-19-related myocarditis, studies are yet inconclusive to follow a regular treatment option in particular. Nevertheless, intravenous immunoglobulins (IVIG) and corticosteroids in combination have been widely used with an effective outcome so far [11]. Our patient received similar medical treatment in addition to antiarrhythmics and beta-blockers with an uneventful hospital stay.

## Conclusion

In conclusion, CMR is a recommended promising tool to address various challenges in the diagnosis of myocarditis and also in post-COVID-19 associated acute myocarditis. Moreover, CMR enables the noninvasive diagnosis of myocarditis providing an excellent diagnostic accuracy with improved acquisition protocol together with the introduction of advanced mapping techniques.

## CRediT authorship contribution statement

Please specify the contribution of each author to the paper, e.g. study design, data collections, data, analysis, writing, others, who have contributed in other ways should be listed as contributors.Dr. Nirmal Prasad Neupane- study design and analysis, Dr. Kritisha Rajlawot – manuscript preparation, Dr. Chandramani Adhikari – manuscript preparation, Dr. Devraj Kandel – Figure collection and preparation, Dr. Irfa Mustafa – Figure collection and preparation.
